# Single-Cell Isolation Microfluidic Chip Based on Thermal Bubble Micropump Technology

**DOI:** 10.3390/s23073623

**Published:** 2023-03-30

**Authors:** Chao Xu, Kun Wang, Peng Huang, Demeng Liu, Yimin Guan

**Affiliations:** 1 School of Microelectronics, Shanghai University, Shanghai 201800, China; 2 Shanghai Aure Technology Limited Company, Shanghai 201800, China

**Keywords:** single-cell isolation, microfluidic chip, thermal bubble micropump

## Abstract

The isolation of single cells is essential for the development of single cell analysis methods, such as single-cell sequencing, monoclonal antibodies, and drug development. Traditional single-cell isolation techniques include flow cytometry (FACS), laser capture microdissection (LCM), micromanipulation, etc., but their operations are complex and have low throughput. Here, we present a microfluidic chip that can isolate individual cells from cell suspension and release them onto a well plate. It uses thermal bubble micropump technology to drive the fluid flow, and single-cell isolation is achieved by matching the flow resistance of the flow channel. Therefore, injection pumps and peristaltic pumps are not required for cell loading. Because of its small size, we can integrate hundreds of single-cell functional modules, which makes high-throughput single-cell isolation possible. For polystyrene beads, the capture rate of the single bead is close to 100%. Finally, the method has been applied to cells, and the capture rate of the single cell is also about 75%. This is a promising method for single-cell isolation.

## 1. Introduction

The isolation and analysis of single cells is an emerging field in the life sciences [1]. The cell is the basic unit of a living organism. Although the cell population has obvious synchronization, the results of single-cell analysis show that even the same cell line or tissue will exhibit differences in gene transcription and translation, protein activity, and metabolite abundance during cell division and differentiation, namely intercell heterogeneity [2]. The observed heterogeneity may be caused by genetic changes, local differences in the cellular environment, or simply by chance (such as the randomness of regulatory events) [3]. Traditional cell-based assays measure the average response of a cell population and study cells at the population level, assuming that the average response is representative of each cell. However, the average response often just reflects the number of cells that are dominant [4]. In doing so, important information about a small but potentially related subpopulation can be lost, especially if that subpopulation determines the behavior of the entire population. For example, the tumor microenvironment is a complex, heterogeneous system consisting of multiple complex interactions between tumor cells and their adjacent noncancer stromal cells. Stromal cells are composed of endothelial cells, fibroblasts, macrophages, immune cells, and stem cells. Due to differences in genetic and environmental factors, different kinds of cells have unique behaviors and have different meanings in disease-causing conditions [5]. Single-cell analysis plays an important role in analyzing the heterogeneity between cells and exploring the differences between individual cells. It can help us deepen our understanding of the cell state, the genome, and even disease pathology [6]. Single-cell analysis plays an important role in drug screening, tissue and organ regeneration, stem cell research, and single-cell sequencing [7].

Single-cell isolation is a prerequisite and important step in single-cell analysis, such as when a developing embryo, brain, or tumor has a complex structure consisting of many types of cells that can be spatially separated. The isolation of different cell types is therefore essential for further cell analysis and of great value in diagnostics, biotechnology, and biomedical applications [8]. At present, it is not easy to isolate a large number of intact single cells with good activity. Traditional single-cell isolation techniques include flow cytometry (FACS), laser capture microdissection (LCM), micromanipulation, immunomagnetic beads, limited dilution, etc. [9]. The limited dilution technique is not accurate for separating single cells. LCM provides a fast and reliable method of obtaining desired target cells from a wide range of cell and tissue preparations through microscopic visualization [10]. Unlike LCM, which mainly isolates single cells from fixed tissue sections, micromanipulation plays an important role in the isolation of live cultured cells or embryonic cells [11]. However, the single-cell isolation throughput is low and requires high operator expertise. FACS has superior enrichment and screening capabilities, but requires a large number of suspended cells (more than 10,000) and is not suitable for rare cell populations. Moreover, the experimental equipment for FACS is usually complex and expensive [12]. Microfluidic technology, developed in the 1990s, greatly promoted the development of single-cell isolation technology, and microfluidic technology has the advantages of miniaturization, high throughput, parallelism, portability, automation, easy operation, low cost, and so on. Microfluidic technology provides a new research platform for single-cell research that can accurately manipulate and analyze microsamples [13,14]. However, traditional microfluidic technology relies heavily on external drive pumps, microvalves, and other devices. The bulky and inconvenient external drive pumps greatly affect the portability of microfluidic technology, and will also increase the requirements for operators. The advantages and disadvantages of the various single-cell isolation techniques are shown in Table 1. The isolation of different cell types is therefore crucial for further cell analysis and will be of great value for diagnostics, biotechnology, and biomedical applications [8].

Microfluidic technology reduces the size of the flow channel to the micron level and matches the diameter of common mammalian cells (10 µm to 20 µm), which is conducive to single-cell operation. Because of its simple microfluidic technology, it has the advantages of low measurement cost, low reagent consumption, accurate cell processing, and so on. It also achieves micro-efficient single-cell isolation and analysis by precisely controlling the fluid movement [15,16,17,18,19,20]. Based on this, we studied a high-throughput single-cell isolation and automatic release microfluidic chip based on a thermal bubble micropump, taking advantage of the fact that microfluidic technology is easy to combine with other technologies. The microfluidic chip is integrated with a thermal bubble micropump to drive the liquid flow so as to eliminate the need for an external power device and improve the portability and integration of the microfluidic chip. Based on the flow resistance matching of fluid mechanics, the cell capture structure was designed to realize the automatic isolation of the single cell with high throughput, and the velocity of the flow has little effect on the structure. The liquid inlet module is designed to realize convenient filling and reduce the interference of impurities [21]. By optimizing the conditions of capture, we achieved a nearly 100% capture rate of polystyrene beads, which we finally successfully applied to cells.

## 2. Materials and Methods

### 2.1. Reagents

Heat-generating films were obtained from Shanghai Industrial µTechnology Research Institute (Shanghai, China). Polystyrene beads were purchased from Tianjin BaseLine. Phosphate-buffered saline (PBS, 10 mM, pH = 7.4) was purchased from Beijing Solarbio Science & Technology (Beijing, China). CHO cell lines were purchased from the Cell Bank (Shanghai Institutes for Biological Sciences, Shanghai, China); 4% paraformaldehyde and 75% ethanol solution were purchased from Sinopharm (Beijing, China). Bovine serum albumin (BSA) was obtained from the Shanghai University of Traditional Chinese Medicine (Shanghai, China).

### 2.2. Microfluidic Chip Design

**Figure 1:**
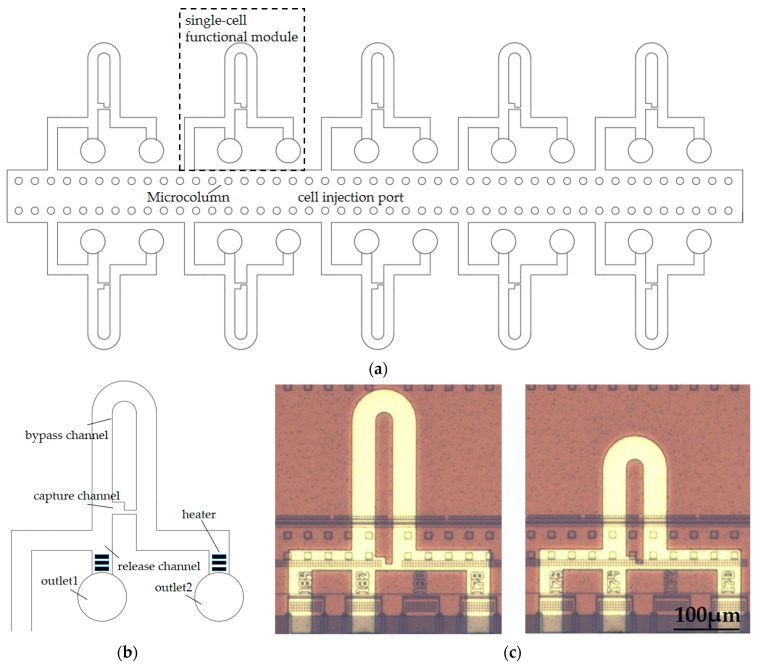
Integrated microfluidic chip for single-cell isolation and release. (**a**) A schematic diagram of the microfluidic chip’s three components (cell injection port, single-cell functional modules, and microcolumn array); (**b**) A single-cell functional module for cell capture and release; (**c**) Photographs of the two different channels.

The structure of the microfluidic chip is shown in Figure 1. The single-cell sorting microfluidic chip mainly consists of a cell injection port, single-cell functional modules, and a microcolumn array (Figure 1a). Microcolumn arrays are used to intercept impurities and multicellular clumps, preventing large volumes of impurities and multicellular clumps from entering single-cell capture channels and causing blockage. Each single-cell capture module consists of four parts (Figure 1b), which are the capture channel, bypass channel, release channel, and outlets 1 and 2, which are formed by thermal bubble micropumps. When there are no cells in the capture channel, the cells in the fluid flow preferentially to the capture channel with the least flow resistance. When the cells are captured by the capture channel, the following cells will automatically flow from the bypass channel to outlet 2 instead of blocking the capture channel. The captured cells are released from outlet 1 by controlling the thermal bubble micropump.

Here, we designed two different bypass channel lengths to investigate the effect of different flow resistance ratios on single-cell capture. The detailed diagram of the microfluidic chip is shown in Figure 1c.

### 2.3. Thermal Bubble Micropump

**Figure 2:**
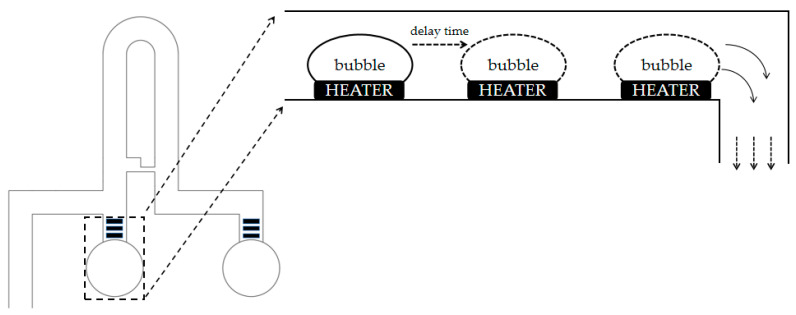
The thermal bubble micropump continuously produces thermal bubbles through the heater, and liquid flows under the action of thermal bubbles.

A thermal bubble micropump was made using micro/nano processing technology and installed at the bottom of the microchannel. The thermal bubble micropump, driven by substrate CMOS circuits, applies a voltage pulse to the heater, which vaporizes the liquid above, forming a thermal bubble at an instantaneous high temperature [22]. After the former heater generates a thermal bubble, there is a delay, and the latter heater generates a thermal bubble, pushing the liquid to flow. After the first thermal bubble is generated, it pushes the liquid to flow. There is a delay before the first thermal bubble disappears and the second thermal bubble is generated, and the liquid can only flow downstream because the first thermal bubble increases the flow resistance upstream. Thermal bubbles are produced in turn, thereby driving the directional flow of the liquid. Each single-cell functional module consists of two thermal bubble micropumps with the same design but different functions. The thermal bubble micropump at outlet 2 is used to drive the cell suspension flow, and the thermal bubble micropump at outlet 1 is used for single-cell output to transfer the captured single cells to the designated container for subsequent analysis. The cross section of the thermal bubble micropump is shown in Figure 2.

### 2.4. Microfluidic Chip Fabrication

**Figure 3:**
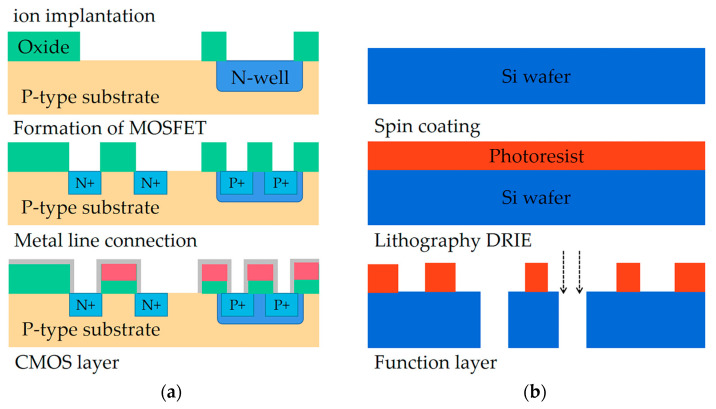
Fabrication of the integrated microchip. (**a**) Manufacturing flow diagram of CMOS layer. (**b**) Manufacturing flow diagram of function layer.

The microfluidic chip was manufactured using Micro-Electro-Mechanical System (MEMS) technology, as outlined in Figure 3. For the CMOS layers, we adopted the n-well process. First, we performed ion implantation on the P substrate to ensure a consistent substrate concentration in batches, and then we laid a photoresist layer on the oxide to produce field oxide and gate oxide by photolithography. Then polycrystalline silicon was generated by dry etching as a gate, and ion implantation was performed to generate the N-well and the source and drain of NMOS. The next step was to etch out the wiring holes to complete the metal connection.

For the function layer, first, the substrate was covered with a layer of film, and the surface of the film was evenly attached with a layer of light glue by the glue machine. Then the image on the mask was transferred to the photoresist layer, and the microchannel structure image was produced by photolithography. Then the image from the lithography was transferred to the film, and the etching was completed on the substrate to produce a certain depth of microchannel structure.

For the thermal bubble micropump, a row of heat-generating films (heater) was placed on the silicon substrate. Then, through dry etching, two holes were formed on the silicon surface as outlet 1 and outlet 2.

### 2.5. Microchannel Pretreatment

**Figure 4:**
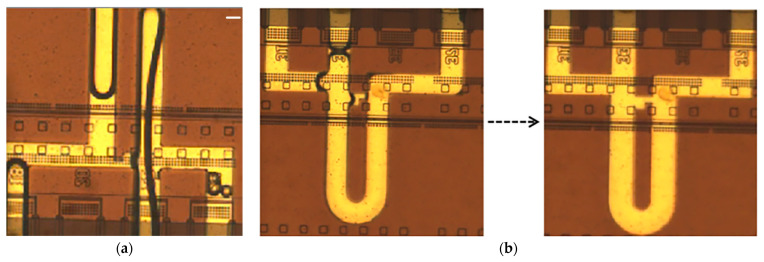
A comparison of the flow channel without pretreatment and the flow channel with pretreatment. (**a**) The flow channel without pretreatment produced large bubbles after the cell suspension was added, which affected the conduct of the experiment. (**b**) A diagram of microchannel preprocessing: after adding 75% ethanol solution to the runner, the runner is quickly soaked in 75% ethanol solution, and no bubbles are produced.

The function of this microfluidic chip to capture single cells is based on the matching of flow resistance. The flow resistance of the capture channel is smaller than that of the bypass channel, so the cells flowing through the flow channel have a high probability of being captured. This can cause a problem. During the first refueling, the fluid will also flow preferentially from the capture channel to outlet 2, while the fluid flowing to the bypass channel has not yet reached outlet 2. This causes bubbles to form in the bypass channel (Figure 4a), and, due to the resistance of the bubbles, subsequent incoming liquid will only flow from the capture channel to outlet 2. If cell suspension is added at this time, cells will accumulate in the capture channel, which will not only affect the capture rate of the single cell but also cause the blockage of the flow channel.

Therefore, it is necessary to pretreat the microchannel, and we chose to use 75% ethanol solution as a lubricant. As a common lubricant, 75% ethanol solution can reduce the surface tension of water, improve the fluidity of liquids, and promote the formation of a water film, thus contributing to the uniform wetting of the flow channel. Before adding the cell suspension to the flow channel, we pretreated the channel by adding the 75% ethanol solution. After the 75% ethanol solution entered the flow channel, the infiltration effect on the flow channel was as shown in Figure 4b. Then we used the thermal bubble micropump to drive the liquid flow to release the 75% ethanol solution from the flow path while constantly adding deionized water. After repeated dilution with deionized water, we assumed that the 75% ethanol solution had been completely removed. This allowed us to add a cell suspension to the flow channel and start the single-cell isolation experiment.

### 2.6. Cell Treatment

CHO cells were removed from culture dishes, mediated by trypsin, and centrifuged at 1500 RPM for 10 min. After fixation with 4% paraformaldehyde, the cells were blocked with 1% BSA to prevent nonspecific binding and were resuspended in PBS.

## 3. Results and Discussion

### 3.1. Mechanism of Single-Cell Functional Chip

**Figure 5:**
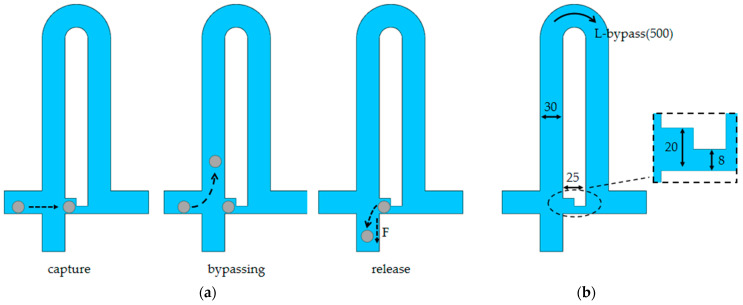
Mechanism of single-cell sorting. (**a**) A schematic diagram showing cell capture, bypassing, and release; (**b**) the specific dimensions of the isolation structure.

The working principle of the cell capture unit is shown in Figure 5a. In order to better capture cells, a bypass channel was designed to allow only a single row of cells to pass through; it has greater flow resistance than the capture channel. When one cell blocks the capture channel, its flow resistance increases dramatically, directing the following cells to the bypass channel discharged via outlet 2, ensuring single-cell capture. This microfluidic chip structure has release channels and outlets that are combined with the thermal bubble micropump. These outlets allow captured cells to be released from the capture channel as needed, and by controlling the thermal bubble micropump at different locations, they flow to the designated outlet.

Based on previous studies, we set 8 µm as the minimum gap in the capture channel [23,24] and designed the capture location to accommodate only one cell with a width of 20 µm (Figure 5b). The capture channel has a length of 25 µm. When flow resistance matching is performed, the length and area of the bypass channel can be reduced. Due to manufacturing errors, the width of the real object is smaller than the design. In order to ensure a single row of cells and prevent clogging, the width of the bypass channel is 30 µm and the height is 20 µm. Because cells cannot completely block the capture channel, when the flow resistance of the capture channel is much smaller than that of the bypass channel, it is easy to cause multicellular capture [25]. Therefore, according to COMSOL Multiphysics Simulation, the length of the bypass channel is 500 µm. In this case, the flow resistance of the captured channel is slightly smaller than that of the bypass channel. A bypass channel length of 300 µm was designed to compare with this structure. In this case, the flow resistance through the bypass channel is smaller than that of the capture channel.

### 3.2. Numerical Simulation of the Single-Cell Functional Module

In order to better understand the flow characteristics around the capture channel and determine the size of the channel, a numerical simulation of the microfluidic chip was carried out using COMSOL Multiphysics software. A 3D laminar flow model based on the steady-state Navier–Stokes’ equation for fluid flow was used for the simulation. The incompressible flow model was used to select water as a fluid, with a density of 1000 kg/m³ and a dynamic viscosity of 0.001 pa·s. The boundary conditions of 300 µm/s and zero pressure were used at the inlet and outlet, respectively, and no-slip conditions were applied to all walls.

**Figure 6:**
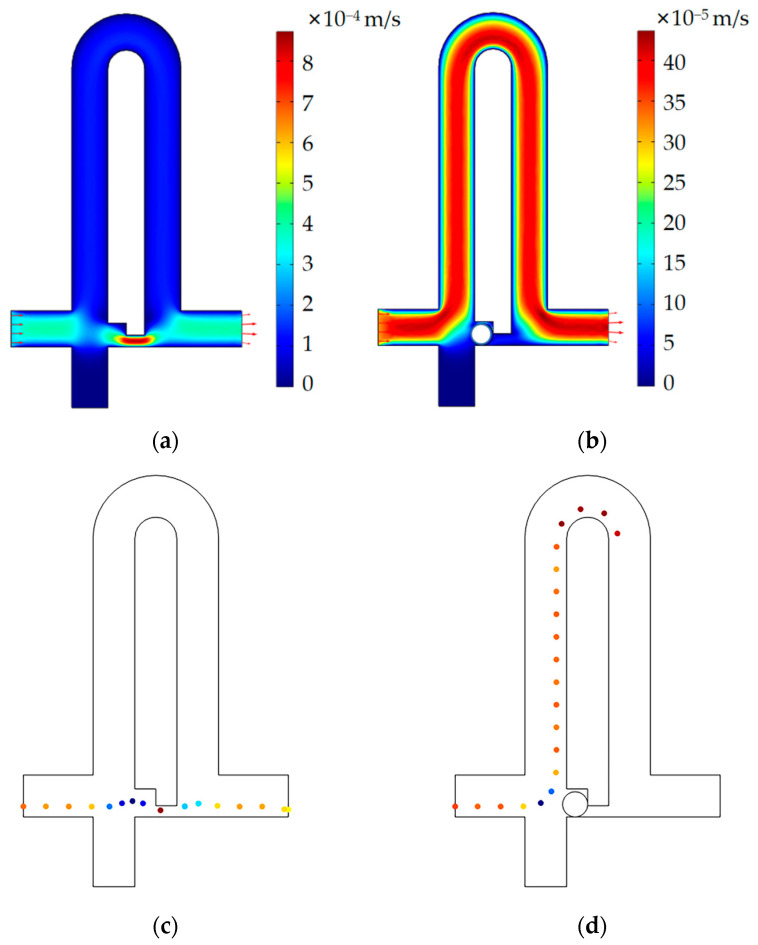
The function of the isolation module is verified by numerical simulation. (**a**) The velocity profile of a single-cell functional module without cell capture; (**b**) the velocity profile of a single-cell functional module with cell capture. (**c**) When the capture channel is vacant, the particles preferentially flow to the capture channel because the flow resistance of the capture channel is smaller than that of the bypass channel. (**d**) When the capture channel captures the cell, the flow resistance of the capture channel suddenly increases, and the particles preferentially flow to the bypass channel.

The velocity plane diagram obtained according to the size of the 500 µm bypass channel above is shown in Figure 6. The velocity distribution is shown at the central cross section of the microfluidic chip, and the velocity of the capture channel is at its maximum, as shown in Figure 6.

Less flow resistance means a larger flow flux, which can be calculated by a surface integration instrument. When the bypass channel is 500 µm, the flow flux of the capture channel (9.25 × 10^−14^ m³/s) is larger than that of the bypass channel (7.81 × 10^−14^ m³/s), which is consistent with the design philosophy. When the capture channel is occupied, the flow velocity of the bypass channel is the largest (Figure 6b), and the flow flux (1.13 × 10^−13^ m^3^/s) is larger than that of the capture channel (5.78 × 10^−14^ m^3^/s). Therefore, the size of the single-cell functional module is feasible for single-cell capture. When the bypass channel is 300 µm, the simulation results show that the flow flux of the bypass channel (1.08 × 10^−13^ m^3^/s) is greater than that of the capture channel (6.39 × 10^−14^ m^3^/s).

In order to more intuitively understand the flow direction of cells in the single-cell functional module after the cell suspension enters the flow channel, we used the particle tracking model for fluid flow in COMSOL Multiphysics software to simulate the particle trajectories. We set the diameter of the particle to 15 µm, depending on the size of the target cell, and the density of the particles and fluid was 1000 kg/m³. All the other conditions were consistent with the above. Consistent with our design, when the capture channel is vacant, particles will preferentially flow from the capture channel to the outlet; when the capture channel is occupied, particles will flow from the bypass channel to the outlet. The simulation results of particle tracking are shown in Figure 6c,d.

### 3.3. Optimization of Capture Conditions

Three different concentrations (C1: 8 × 10^3^ per mL, C2: 4 × 10^4^ per mL, C3: 8 × 10^4^ per mL) of polystyrene beads were used to verify the single-cell functional module. The 14 µm polystyrene bead group was a suitable model to characterize the system [1].

**Figure 7:**
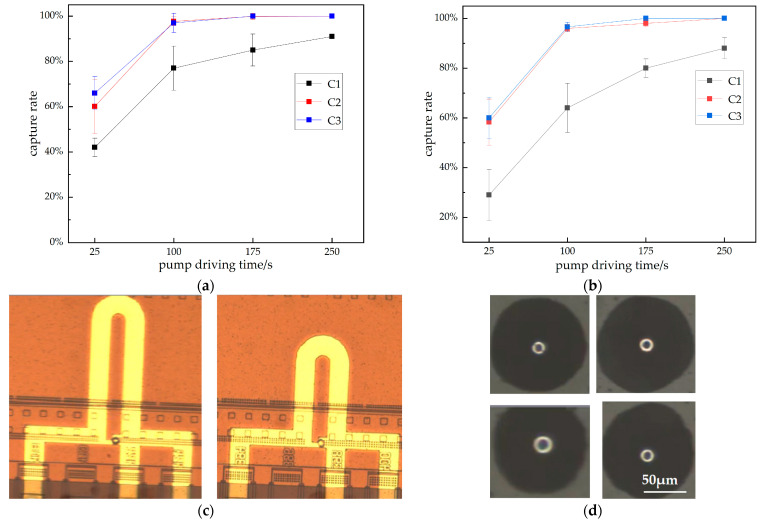
The relationship between the capture rate of microspheres with different concentrations and pump driving times. The longer the micropump’s driving time, the more liquid the capture module flows through. This means more cells flow through the trapping module. (**a**) Capture rate curve of the long bypass channel; (**b**) capture rate curve of the short bypass channel; (**c**) image of a microfluidic chip that captured beads; (**d**) image of the bead released on the slide.

The influence of the pump’s driving time on the capture rate is shown in Figure 7. We defined the capture rate as the number of units captured by the bead over all units. The trend and direction of the curves were the same for both structures. For the same concentration, the capture rate increases with the pump’s driving time. For the same pump driving time, the capture rate increases with the concentration. For C2 and C3, the difference between the concentration and pump driving time curves is not very large. This indicates that, when the concentration reaches a certain level, increasing the concentration has little effect on the capture rate. When the pump’s driving time reaches 100 s, the capture rate increases slowly as the pump’s driving time increases. For C1, despite a lengthy pump drive time, the capture rate cannot reach 100%. This could be due to the low concentration of beads, along with a certain amount of polystyrene bead loss.

By comparing Figure 7a,b, it is found that the capturing ability of the long channel is stronger than that of the short channel. This is consistent with our theory. For the captured beads, we can release them from outlet 1, and the physical image of the bead released on the slide is shown in Figure 7d.

### 3.4. Comparison of the Capture Rate of the Single Bead with Two Structures

**Figure 8:**
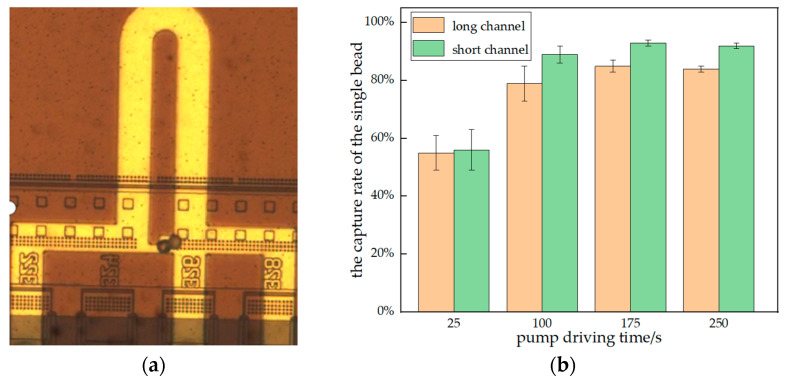
(**a**) A capture channel that trapped two beads; (**b**) the capture rate of the single cell. This shows the effect of the length of the bypass channel on bead multiple capture. Bead multi-capture was significantly lower with a short channel structure.

During the experiment, it was found that, with the increase in concentration of polystyrene beads, the multiple capture of beads also increased, is shown in Figure 8a. To compare the performance of the two structures in single-cell capture, we recorded the capture rates of the single bead in both structures at a concentration of 8 × 10^4^ beads per mL. The comparison of the capture rates of single beads by the two structures is shown in Figure 8b.

According to the above experimental results, the capture rate of beads with a long channel is higher than that with a short channel at the same concentration. However, as Figure 8b shows, the capture rate of the single bead in short channels was higher than that in long channels. This indicates that, although the capturing ability of the long channel structure is stronger than that of the short channel structure, it is also more likely to result in multiple captures of beads. The short channel structure performs poorly in terms of capturing but excels in terms of capturing a single bead. Therefore, without consideration of cells, we can consider using a shorter bypass channel structure in exchange for a higher capture rate of a single cell.

### 3.5. Cell Experiment

**Figure 9:**
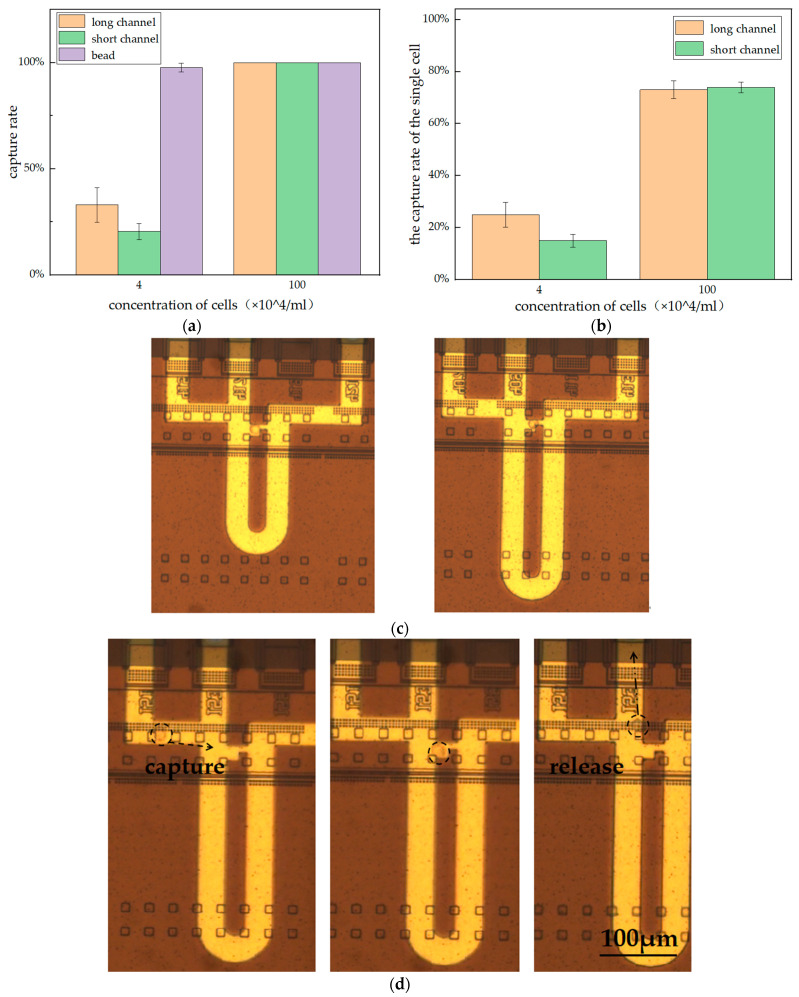
(**a**) Comparison of capture rates at two different cell concentrations; on the far right is the capture rate of the beads at the same concentration. (**b**) Multicellular capture occurred frequently at high concentrations; (**c**) image of a microfluidic chip that captured a single cell; (**d**) the process of cell capture, from capture to release.

Through the above experiments, an optimized cell capture condition was obtained. We used CHO cells fixed with 4% paraformaldehyde and suspended in PBS. The cell concentration is 4 × 10^4^ cells per mL, and the pump driving time is 100 s. Under this condition, the capture rate of cells was much smaller than that of beads (Figure 9a). It was observed that some cells were squeezed out of the capture channel during liquid flow due to their soft texture.

When the concentration of cells reached 10^6^ cells per mL, the capture rate reached 100%. This means that, when the cell concentration is sufficient, a capture rate of 100% can be achieved, as shown in Figure 9b. However, the increase in cell concentration inevitably leads to more multicellular capture and more cells being depleted. The capture rate of the single cell at two different concentrations is shown in Figure 9b. At a cell concentration of 10^6^ cells per mL, the capture rate of the single cell in both long and short channels was around 75%. That means that a quarter of the channels capture more than one cell. We believe that optimizing the capture structure based on the cellular soft texture can improve the capture rate of single cells.

The microfluidic chip’s capability to successfully capture a single cell is shown in Figure 9c, and the process of cell capture and release is shown in Figure 9d.

## 4. Conclusions

The microfluidic chip can capture single cells by matching the flow resistance of the capture channel and the bypass channel. The thermal bubble micropump provides the driving force for cell suspension flow and cell export without the need for external power sources such as injection pumps. Because of its small size, we can integrate hundreds of single-cell functional modules, which makes high-throughput single-cell isolation possible. The microfluidic chip is fabricated by mature semiconductor processing technology with high integration, which provides a guarantee of a high throughput of single-cell sorting. The capture rate of polystyrene beads reached nearly 100%. Finally, the method was applied to cells, and the capture rate of a single cell was also about 75%. In future work, for our microfluidic chip to be more versatile, we will improve the size of the functional module channel according to the size of the target cell. Our microchip is currently being further optimized for monoclonal antibody development and is a very promising single-cell isolation chip.

## Figures and Tables

**Table 1:** Comparison of various techniques for single-cell isolation [9].

Properties	Micromanipulation	FACS	LCM	Other Microfluidics	Our Technology
Throughput	Low	High	Low	High	High
Starting number of cells	Low	High	Low	Low	Low
Operational complexity	High	Normal	High	Low	Low
Portability	Low	Normal	Low	Normal	High
Others	Low efficiency and long collection time	Expensive equipment and cell dissociation	May compromise RNA quality	Relies heavily on external drive pumps	No external drive pumps are required

## Data Availability

The data sets used or analyzed during the current study are available from the corresponding author on reasonable request.
